# Zinc-L-Carnosine (Polaprezinc) in managing infant regurgitation: a two-center randomized controlled trial

**DOI:** 10.3389/fped.2026.1754324

**Published:** 2026-04-21

**Authors:** Marisa Piccirillo, Maurizio Mennini, Enrico Felici, Angela Mauro, Luca Bernardo, Maria Elisabetta Baldassarre, Erika Renzi, Corrado De Vito, Silvia Furio, Marco Graziani, Giovanna Quatrale, Alessandro Ferretti, Giovanni Di Nardo

**Affiliations:** 1 Department of Neurosciences, Mental Health and Sensory Organs (NESMOS), Faculty of Medicine and Psychology, Sapienza University of Rome, Rome, Italy; 2 Pediatric Unit, Sant'Andrea University Hospital, Rome, Italy; 3 Pediatric Unit, Children's Hospital, Azienda Ospedaliera SS Antonio e Biagio e Cesare Arrigo, Alessandria, Italy; 4 Rheumatology Unit, Department of Childhood and Developmental Medicine, ASST Fatebenefratelli-Sacco, Milano, MI, United States; 5 Department of Interdisciplinary Medicine, Neonatology and Neonatal Intensive Care Unit, “Aldo Moro” University of Bari, Bari, Italy; 6 Department of Public Health and Infectious Diseases, Sapienza University of Rome, Rome, Italy; 7 Pediatric Gastroenterology and Endoscopy Unit, Department of Pediatric Specialties, Santobono Pausilipon Children's Hospital, Naples, Italy

**Keywords:** gastroesophageal reflux, infant regurgitation, polaprezinc, thickened formula, Zinc-L-carnosine

## Abstract

**Background:**

Gastroesophageal reflux (GER) is a common physiological phenomenon in infants, typically self-limited, but often a source of parental concern. When non-pharmacological measures fail to improve symptoms, thickened formulas are commonly used. Zinc-L-carnosine (Polaprezinc) is a mucosal protectant with anti-inflammatory and antioxidant properties. It is widely used in gastrointestinal disorders in adults but has not yet been extensively studied in infants. We aim to evaluate the clinical effectiveness and cost impact of a Zinc-L-carnosine-based supplement (Hepilor liquido®) compared to thickened formula in infants with persistent regurgitation.

**Methods:**

This was a two-center, prospective, single-blind, randomized, non-inferiority study conducted in two Italian pediatric hospitals. Infants aged 4 weeks to 7 months with persistent regurgitation despite appropriate nutritional and behavioral management were randomized to receive either Hepilor liquido® or thickened formula for 8 weeks. The primary outcome was symptom improvement based on the I-GERQ-R (Infant Gastro-Esophageal Reflux Questionnaire-Revised) score. Secondary outcomes included a reduction in regurgitation frequency and treatment cost analysis.

**Results:**

Sixty infants aged 4 weeks to 7 months were randomized to receive either Hepilor liquido® (*n* = 30, 50%) or thickened formula (*n* = 30, 50%) for 8 weeks. Both groups showed significant improvement in I-GERQ-R scores at 8 weeks. Although no statistically significant difference was observed in overall symptomatic remission, the Hepilor liquido® group demonstrated a greater reduction in regurgitation frequency. Notably, the average treatment cost was significantly lower in the Hepilor® group compared to the thickened formula. No severe adverse events were recorded in both groups.

**Conclusions:**

Zinc-L-carnosine is a safe and effective alternative to thickened formula in treating persistent infant regurgitation, after the failure of non-pharmacological measures. Its lower cost and promising clinical effect support further investigation in larger pediatric cohorts.

**Clinical Trial Registration:**

https://clinicaltrials.gov/study/NCT06678997, identifier: NCT06678997.

## 1 Introduction

Gastroesophageal reflux (GER) is a common condition in which stomach contents flow back into the esophagus. In healthy infants with normal growth, GER is considered a physiological phenomenon (1). Regurgitation and occasionally vomiting, after most feeds, is reported in approximately 20% of healthy one-month old infants. This prevalence increases to 41% between 3 and 4 months of age, then gradually decreases, becoming rare after the first year of life (2, 3).

In children, several anatomic conditions predispose to GER. For instance, a short intra-abdominal esophageal tract facilitates the upward movement of gastric contents. Additionally, infants consume roughly twice the amount of liquid per kg compared to adults (100–150 mL/kg/day), enhancing the frequency of transient lower esophageal sphincter relaxations (TLESRs). Frequent feeding, numerous naps and post-prandial periods (when TLESRs commonly occur) also contribute to the higher incidence of GER in infants (4).

Typical symptoms of GER in infants include regurgitation, irritability, crying, and sometimes vomiting (5). When regurgitation does not impair growth or cause other complications, it is considered physiological, and such infants are often referred to as “happy spitters.” According to the Rome IV criteria, these cases are diagnosed as functional regurgitation (6). Despite resolving naturally within the first year, functional regurgitation remains a frequent concern for caregivers (7). Consequently, although current guidelines do not recommend them, thickened formulas or alginate-based formulations are usually prescribed to infants with persisting regurgitation (8). Thus, there is a clinical need for safe and effective products for managing GER symptoms in infants.

Polaprezinc is a complex of a zinc ion, L-carnosine, a b-alanine dipeptide, and L-histidine currently used as an antiulcer agent in Japan, that has demonstrated anti-inflammatory, antifibrotic, antioxidant and antiapoptotic effects (9). It has shown promising results in preventing oral mucositis following radiotherapy and high-dose chemotherapy in adult and pediatric patients with head and neck cancer or Hematopoietic Stem Cell Transplantation (HSCT) (10–13). It has also been proposed as a mucosal protectant in the treatment of peptic ulcers for eradicating *Helicobacter pylori* and, more recently, in preventing non-steroidal anti-inflammatory drugs (NSAID)-induced small bowel injury (14–16). In Italy, the only available medical device containing Zinc-L-carnosine (Polaprezinc) in a liquid suspension suitable for infants is Hepilor liquido®. This experimental study aimed to investigate the efficacy of Hepilor liquido® in infants with persistent GER symptoms despite appropriate behavioral and nutritional management, as recommended by the North American Society for Paediatric Gastroenterology, Hepatology and Nutrition (NASPGHAN) and the European Society for Paediatric Gastroenterology, Hepatology, and Nutrition (ESPGHAN) societies.

## 2 Materials and methods

### 2.1 Study design

This was a two-center randomized, controlled, single-blind, non-inferiority trial conducted between November 2024 to April 2025 in infants presenting with regurgitation, referred to two pediatric Italian hospitals. The study was registered at ClinicalTrials.gov on 2025-11-12 (NCT06678997; https://clinicaltrials.gov/study/NCT06678997). The Ethical Committee approved the study and written informed consent was obtained from all participating parents.

### 2.2 Study population

Inclusion criteria were represented by consecutive infants (aged 1 to 7 months) with at least two episodes of regurgitation per day for three or more weeks, according to Rome IV criteria for infant regurgitation. Exclusion criteria were as follows: 1) breastfed infants; 2) infants who already started complementary feeding; 3) presence of retching, hematemesis, aspiration, apnea, failure to thrive, feeding or swallowing difficulties, or abnormal posturing 4) known or suspected metabolic, hepatic, renal or other chronic disease; 5) previous or current use of thickened formulas; 6) previous treatment with alginate or H2-receptor antagonists or proton pump inhibitors (PPIs).

### 2.3 Intervention

All parents entered in a run-in period of two weeks and were taught to adopt a non-pharmacological approach (avoiding overfeeding, incorrect positioning, and passive smoking, in accordance with ESPGHAN guidelines (5) and the National Institute for Health and Care Excellence (NICE) guidelines (17). Parents answered the Infant Gastro-Esophageal Reflux Questionnaire-Revised (I-GERQ-R) questionnaire two weeks after enrolment. The I-GERQ-R is a 12-item multiple-choice questionnaire designed and validated to objectively score and evaluate infant GER-related symptoms and monitor treatment outcomes in clinical practice, as well as an evaluative tool in clinical trials (18). In I-GERQ-R, the frequency and quantity of GER symptoms in the past 7 days are surveyed. A maximum total score of 42 can be achieved, with higher scores pointing towards a larger burden of GER symptoms. We considered persistent regurgitation when I-GERQ-R was above the cut-off limit of normal (≥16), as already reported (8). Only infants with I-GERQ-R ≥ 16 after the run-in period were recruited for the intervention study. Patients were assigned in a single-blinded manner to the intervention or control group according to a computer-generated randomization allocation table. An independent physician not directly involved in the study was responsible for the randomization process. Infants were randomly assigned to receive Hepilor (Group A) or a cornstarch-thickened formula (Group B) orally. Regarding Hepilor, the recommended dose was 2.5 mL twice daily for children weighing <5 kg and 5 mL twice daily for children weighing >5 kg; each dose was administered one hour after each feeding. Group B received thickened formula at 150 mL/kg/day. Parents were also instructed to avoid other treatments, such as probiotics, prokinetics, or acid inhibitors, or start any variation in coping, position, or infant's diet during the study. Anthropometric measures and I-GERQ-R were repeated at the end of the 8 weeks treatment period. The investigators collecting and analyzing I-GERQ-R questionnaires and anthropometric measures were blinded for the period during which the data were collected. The study's primary outcome was the proportion of infants reaching an I-GERQ-R < 16 after treatment. Secondary outcomes included a reduction in regurgitation frequency and treatment cost analysis. We assessed the direct costs of thickened formula and Hepilor. The direct cost was calculated considering the quantity of thickened formula and Hepilor used during the eight weeks, based on the price on the Italian market (on average, 34.90 euro/L for thickened formula, and 24.20 euro for 200 mL of Hepilor syrup). Any significant clinical ad-verse effects or problems in administering the formulation were also recorded.

## 3 Statistical analysis

The sample size was calculated using Powerandsamplesize.com© (2013–2019 HyLown Consulting LLC Atlanta, GA, USA) for comparison of two percentages based on data observed in two previous studies assessing the efficacy of non-pharmacologic and pharmacologic treatments in infants with GER (19, 20). Setting a significance level of 5% and a power of 80%, the sample size requested for each treatment group was 24. Assuming a 20% dropout between screening and randomization, we calculated that a minimum of 60 infants had to be enrolled.

Descriptive statistics were obtained using mean and standard deviation for continuous variables and proportions for categorical and dichotomous variables. The normal distribution of continuous data was assessed using the Shapiro–Wilk test. Between-group comparisons were performed using independent samples t-tests, while within-group comparisons over time were evaluated using paired samples t-tests. To confirm the robustness of the results, non-parametric analyses were also conducted using the Wilcoxon signed-rank test for within-group comparisons. Categorical variables were compared using the *Χ*-square test. All analyses used Stata (StataCorp LLC, 4905 Lakeway Drive, College Station, TX 322, United States), version 18.0. A two-sided *p*-value <0.05 was considered statistically significant.

## 4 Results

### 4.1 Baseline characteristics of participants

**Figure 1:**
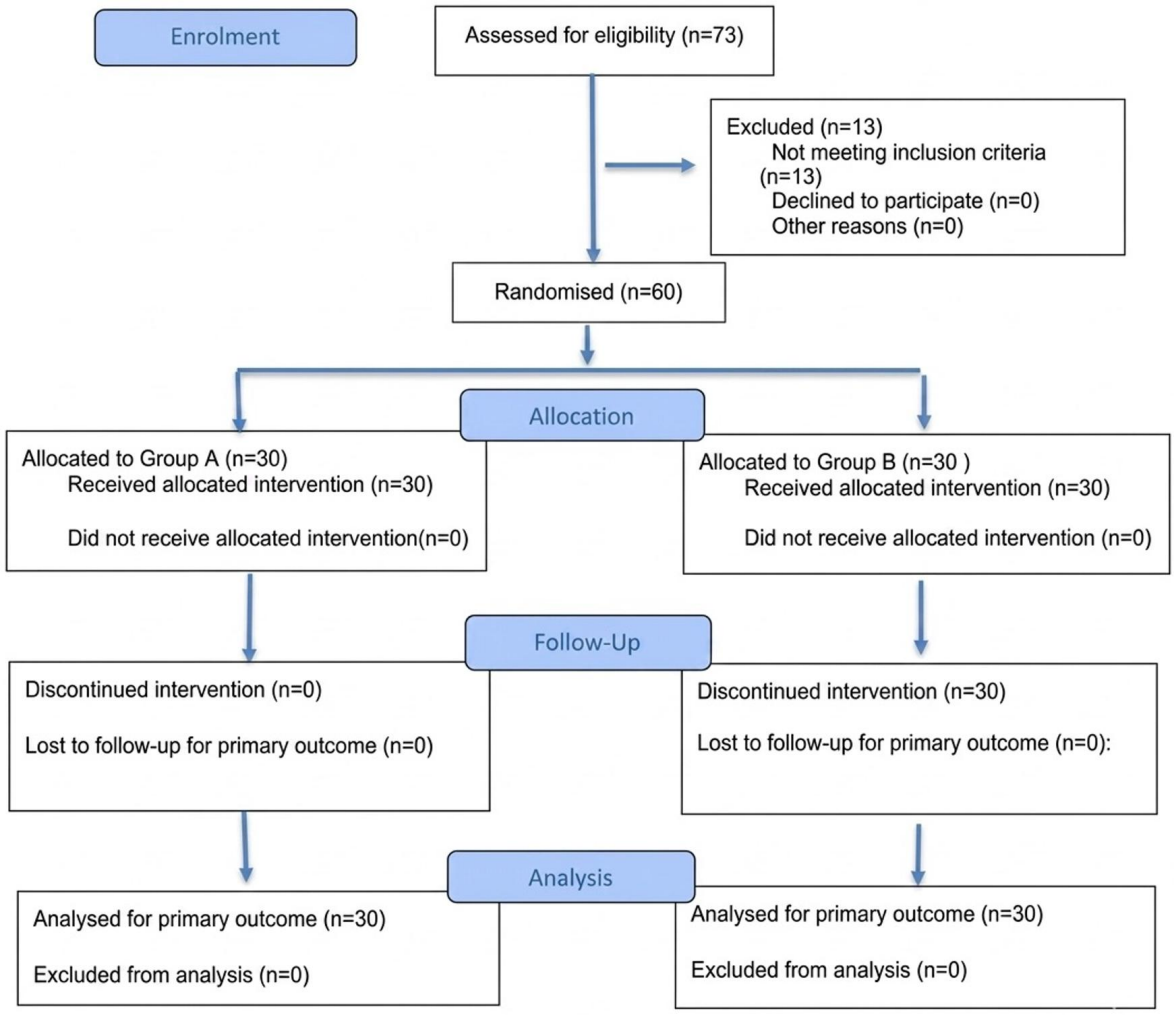
Flow diagram of the progress through the phases of a randomised trial of two groups.

Seventy-three infants satisfied the inclusion criteria. After the two-week run-in period of nutritional and behavioral changes, thirteen infants were excluded for parents reporting a normal I-GERQ-R. Finally, 60 patients were recruited in the intervention phase and were randomly assigned to Group A (*n* = 30, 50%) and Group B (*n* = 30, 50%) (Figure 1). Patients’ baseline and clinical characteristics were not statistically different between the two groups and are reported in Table 1.

**Table 1:** Characteristics of patients in the hepilor (group A) and thickened formula (group B).

Variable	Hepilor (Group A)	Thickened formula (Group B)	*p*-value*
	N (%)	N (%)	
Sex			0.796
Male	16 (53.3)	15 (50.0)	
Female	14 (46.7)	15 (50.0)	
Age in months (mean ± SD)	4.0 (±1.5)	4.03 (±1.1)	0.921

* *p*-value: between-group comparison using independent samples t-test.

### 4.2 Primary outcome

Mean I-GERQ at enrolment was 23.3 ± 2.3 in Group A and 23.4 ± 3.5 in Group B, with no significant differences among groups (*p* = 0.932). Overall, symptomatic remission (I-GERQ-R < 16) was observed both in Group A and in Group B, without statistically significant differences (*n* = 19, 48.7% Group A vs *n* = 20, 51.3% Group B, *p*-value = 0.073) (Table 2).

**Table 2:** Symptomatic remission (I-GERQ-R < 16) in infants treated with hepilor vs thickened formula after 8 weeks of treatment.

Variable	Hepilor (Group A)x(±SD)	Thickened formula (Group B)x(±SD)	*p*-value*
*Symptomatic remission (I-GERQ-R* *<* *16)*			0.073
Yes	19 (48.7)	20 (51.3)	
No	11 (52.4)	10 (47.6)	

* *p*-value: between-group comparison using independent samples t-test.

### 4.3 Secondary outcome

Both groups experienced an improvement in I-GERQ-R scores after 8 weeks of treatment (*p*-value <0.001 within-group comparison), although difference between the two group was not statistically significant (*p* = 0.550) (Table 3). Considering the single items, both treatments show single-item improvement, although with no statistical differences. In reducing the amount of regurgitation (Item 2), Hepilor proved to be significantly more efficient than the thickened formula (1.5 ± 0.51 vs 1.8 ± 0.50, *p*-value = 0.025). However, as for the average time of crying/fussing (Item 8), the T2 score was significantly lower in the thickened formula group than in the Hepilor group (1.2 ± 0.43 vs. 1.6 ± 0.55, *p*-value < 0.001). Details on single-item scores are reported in Table 4.

**Table 3:** Comparison of I-GERQ scores between baseline (T0) and 8 weeks (T2) across treatment groups.

Variable	Hepilor (Group A)x(±SD)	Thickened formula(Group B)x(±SD)	*p*-value^a^
Baseline (T0)	23.3 (±2.3)	23.4 (±3.5)	0.932
8 weeks (T2)	14.8 (±3.2)	14.3 (±3.3)	0.550
*p*-value^b^	<0.001	<0.001	

a *p*-value: between-group comparison using independent samples t-test.

b *p*-value: within-group comparison between T0 and T2 using paired samples t-test.

**Table 4:** Comparison of I-GERQ item scores between baseline (T0) and 8 weeks (T2) across treatment groups.

Variable	Hepilor(Group A)x(±SD)	Thickened formula(Group B)x(±SD)	*p*-value^a^
Item 1 (frequency of regurgitation)			
Baseline (T0)	3.4 (±0.49)	3.3 (±0.46)	0.591
8 weeks (T2)	2.2 (±0.66)	2.2 (±1.8)	1.00
Item 2 (amount of regurgitation)			
Baseline (T0)	3.1 (±0.55)	3.0 (±0.50)	0.519
8 weeks (T2)	1.5 (±0.51)	1.8 (±0.50)	0.025^a^
Item 3 (frequency of discomfort)			
Baseline (T0)	2.9 (±0.51)	2.8 (±0.46)	0.790
8 weeks (T2)	1.9 (±0.58)	1.7 (±0.70)	0.167
Item 4 (feeding refusal)			
Baseline (T0)	2.3 (±0.94)	2.2 (±0.96)	0.787
8 weeks (T2)	1.6 (±0.77)	1.5 (±0.87)	0.747
Item 5 (feeding difficulty)			
Baseline (T0)	2.3 (±0.79)	2.2 (±0.82)	0.749
8 weeks (T2)	1.7 (±0.91)	1.6 (±0.93)	0.577
Item 6 (intensity of crying)			
Baseline (T0)	2.4 (±0.81)	2.4 (±0.77)	0.870
8 weeks (T2)	1.7 (±0.80)	1.6 (±0.89)	0.762
Item 7 (frequency of crying)			
Baseline (T0)	2.1 (±0.45)	2.1 (±0.48)	0.782
8 weeks (T2)	1.7 (±0.66)	1.4 (±0.67)	0.127
Item 8 (duration of crying)			
Baseline (T0)	2.3 (±0.70)	2.4 (±0.72)	0.718
8 weeks (T2)	1.6 (±0.55)	1.2 (±0.43)	<0.001^a^
Item 9 (frequency of fussing)			
Baseline (T0)	0.9 (±0.73)	0.9 (±0.72)	0.856
8 weeks (T2)	0.3 (±0.71)	0.3 (±0.65)	0.851
Item 10 (frequency of arching back)			
Baseline (T0)	1.6 (±0.79)	1.7 (±0.72)	0.734
8 weeks (T2)	0.9 (±0.60)	0.8 (±0.46)	0.550
Item 11 (struggling in breathing or apnea)			
Baseline (T0)	0.4 (±0.57)	0.4 (±0.50)	0.810
8 weeks (T2)	NA^b^	NA^b^	–
Item 12 (presence of cyanosis)			
Baseline (T0)	NA^b^	NA^b^	–
8 weeks (T2)	NA^b^	NA^b^	–

a *p*-value: between-group comparison using independent samples t-test.

b NA: not assessed.

### 4.4 Cost analysis

A mean cost saving of approximately 759.32 euro (36.35%) per child was registered during the 8-week treatment period in Group A (Hepilor) compared to Group B. Universal protocol adherence was observed, with all pediatric participants demonstrating willing engagement with the treatment intervention and no severe adverse events were recorded in both groups.

## 5 Discussion

Current guidelines recommend a nonpharmacological approach in infants with GER, suggesting thickened feed for treating visible regurgitation/vomiting (5). However, thickened feeds are associated with reported resistance to the texture from the child and increased viscosity, which affects the feeding time (21, 22). Likewise, current European guidelines state that it is uncertain whether using alginates may improve signs and symptoms of GER based on the I-GERQ-R questionnaire. Our study suggests that infants with persistent regurgitation, not improving after behavioral and feeding advice, may benefit equally from thickened formula and Hepilor supplementation. Polaprezinc [N-(3-aminopropionyl)-L-histadinazinc] is a new generation gastric mucosal protective agent, consisting of zinc ion and L-carnosine (9). It provides mucosal protection by increasing heat shock protein expression (23–25), inhibiting the expression of inflammatory factors such as Tumor Necrosis Factor (TNF)-alpha and Nuclear Factor (NF)-kB, and holding antiapoptotic and antioxidant effects (26, 27). It is also capable of inducing proliferation and migration of granulation tissue in injured epithelial cells (10). Polaprezinc has also proven effective in treating peptic ulcers in adults (28) and in preventing oral mucositis following high-dose chemotherapy in adult and pediatric patients before HSCT (13), as well as in adult patients with head and neck cancer receiving radiotherapy (10–12).

In our study, symptomatic remission, defined as I-GERQ-R < 16 after 8 weeks of treatment, was observed in both groups without statistically significant differences (*n* = 19, 48.7% Group A vs. *n* = 20, 51.3% Group B, *p*-value = 0.073). Our study also showed that both groups experienced improvement in I-GERQ-R scores after 8 weeks of treatment.

Symptomatic remission after the use of thickened formula has already been proven, although only two studies evaluated treatment efficacy through a validated questionnaire (29, 30). Conversely, the efficacy of Polaprezinc on symptomatic remission in infants with GER has never been investigated before. The effectiveness of the thickened formula can be attributed to the increased viscosity of gastric contents, which physically reduces the likelihood of refluxed material rising through the lower esophageal sphincter (22). On the other hand, Zinc L-Carnosine forms a protective layer on the gastric and esophageal mucosa. It benefits from a relative site selectivity, slow dissociation rate and acid-independent mechanism of action that may reduce damage from reflux and thus improve GER symptoms (31). Polaprezinc does not affect acid secretion in the mucosa when given intragastrically, therefore suggesting that the mechanism of mucosal protection is not mediated by acid suppression (32). This approach may appear particularly advantageous in cases of predominantly non-acidic reflux, representing the prevalent form in infants, as highlighted by Salvatore et al. (33).

In the present study, parents of infants receiving Hepilor supplementation reported a reduction in regurgitation after 8 weeks of treatment compared to the control group (1.5 ± 0.51 vs 1.8 ± 0.50, *p*-value = 0.025). Since inflammatory mediators and other factors have been demonstrated to decrease the lower esophageal sphincter pressure (LESP) and increase the number of transient lower esophageal sphincter relaxation (TLESR) (34), the anti-inflammatory and anti-oxidative properties of Zinc L-Carnosine may explain the observed reduction in the amount of regurgitation.

Our study also demonstrated a significant cost saving when using Hepilor in association with normal formula compared to thickened formula. This finding is consistent with a previous Italian study in which thickened formula proved more expensive than alginates when treating GER in infants (29). Parents often prepare the home-brewed thickened standard formula because of the higher (1.5–2 times) cost of the anti-reflux formula. However, they may add quantities of starch above the regulatory limit, thus increasing the osmolarity of the formula and providing extra caloric intake. In addition, over-thickening of formula results in a higher viscosity that requires increased sucking effort (22). Treating GER in infants with Polaprezinc may be preferable regarding cost savings while providing the same symptom improvement as a thickened formula.

The sample size was calculated to assess the non-inferiority of Polaprezinc compared to the thickened formula with respect to symptomatic remission. Therefore, the study is not powered to detect statistically significant differences between multiple individual items. Additionally, the 2-month follow-up, while aligned with current clinical practice, does not allow for the evaluation of long-term effects of treatments, an aspect that would merit further investigation.

## 6 Strengths and limitations

We acknowledge that the open-label nature of the study introduces potential bias, as parents were aware of which treatment their infant received due to the obvious physical differences between thickened formula and liquid supplement. Since symptom assessment relied primarily on parental reporting, subjective outcomes may have been influenced by parental expectations or perceptions about treatment efficacy. However, we note that: both treatment groups received active interventions with established mechanisms, potentially reducing expectation bias, the use of validated symptom questionnaires provided some standardization, and the similarity in clinical effectiveness between groups suggests that any bias did not dramatically favor one treatment over the other.

Future studies should focus on identifying predictive factors for response to different treatments to personalize the therapeutic approach. It would also be interesting to evaluate the effects of combination therapy (Hepilor associated with thickened formula) in more resistant cases, a hypothesis never explored in the scientific literature.

## 7 Conclusions

In this two-center, randomized study, supplementation with Zinc-L-carnosine (Hepilor liquido®) showed comparable efficacy to the thickened formula in improving symptoms of infant regurgitation, as measured by the I-GERQ-R score. Although no statistically significant difference was found in overall symptomatic remission between groups, Hepilor demonstrated a greater reduction in the quantity of regurgitation and was associated with significantly lower treatment costs. These findings suggest that Zinc L-Carnosine may represent an effective and economically advantageous alternative for the management of persistent GER symptoms in infants after the failure of non-pharmacological measures. Larger, longer-term studies are needed to confirm these results and explore their use in combination strategies or specific subgroups of patients.

## Data Availability

The raw data supporting the conclusions of this article will be made available by the authors, without undue reservation.
